# Blood Test for Breast Cancer Screening through the Detection of Tumor-Associated Circulating Transcripts

**DOI:** 10.3390/ijms23169140

**Published:** 2022-08-15

**Authors:** Sunyoung Park, Sungwoo Ahn, Jee Ye Kim, Jungho Kim, Hyun Ju Han, Dasom Hwang, Jungmin Park, Hyung Seok Park, Seho Park, Gun Min Kim, Joohyuk Sohn, Joon Jeong, Yong Uk Song, Hyeyoung Lee, Seung Il Kim

**Affiliations:** 1 Department of Biomedical Laboratory Science, College of Health Sciences, Yonsei University, Wonju 26493, Korea; 2 Department of Surgery, Yonsei University College of Medicine, Seoul 03722, Korea; 3 Department of Clinical Laboratory Science, College of Health Sciences, Catholic University of Pusan, Busan 46252, Korea; 4 Avison Biomedical Research Center, Yonsei University College of Medicine, Seoul 03722, Korea; 5 Department of Medical Oncology, Yonsei University College of Medicine, Seoul 03722, Korea; 6 Department of Surgery, Gangnam Severance Hospital, Yonsei University College of Medicine, Seoul 06273, Korea; 7 Division of Business Administration, College of Government and Business, Yonsei University, Wonju 26493, Korea

**Keywords:** blood test, breast cancer, early diagnosis, prognosis, tumor-associated circulating transcripts assay

## Abstract

Liquid biopsy has been emerging for early screening and treatment monitoring at each cancer stage. However, the current blood-based diagnostic tools in breast cancer have not been sufficient to understand patient-derived molecular features of aggressive tumors individually. Herein, we aimed to develop a blood test for the early detection of breast cancer with cost-effective and high-throughput considerations in order to combat the challenges associated with precision oncology using mRNA-based tests. We prospectively evaluated 719 blood samples from 404 breast cancer patients and 315 healthy controls, and identified 10 mRNA transcripts whose expression is increased in the blood of breast cancer patients relative to healthy controls. Modeling of the tumor-associated circulating transcripts (TACTs) is performed by means of four different machine learning techniques (artificial neural network (ANN), decision tree (DT), logistic regression (LR), and support vector machine (SVM)). The ANN model had superior sensitivity (90.2%), specificity (80.0%), and accuracy (85.7%) compared with the other three models. Relative to the value of 90.2% achieved using the TACT assay on our test set, the sensitivity values of other conventional assays (mammogram, CEA, and CA 15-3) were comparable or much lower, at 89%, 7%, and 5%, respectively. The sensitivity, specificity, and accuracy of TACTs were appreciably consistent across the different breast cancer stages, suggesting the potential of the TACTs assay as an early diagnosis and prediction of poor outcomes. Our study potentially paves the way for a simple and accurate diagnostic and prognostic tool for liquid biopsy.

## 1. Introduction

Breast cancer accounts for 30% of all oncological diagnoses of women worldwide, and a fifth of breast cancers recur as incurable, direct metastases [1]. According to the World Health Organization, 2.1 million new cases and 627,000 deaths of breast cancer occurred in 2018. Breast cancer is a heterogeneous disease classified into four subtypes by protein expression in tissues, including luminal A (estrogen receptor (ER) or progesterone receptor (PR)+, human epidermal growth factor receptor 2 (HER-2)−, or Ki67+ < 20%), luminal B (ER or PR+, HER-2+, or Ki67+ ≥ 20%), HER-2 (ER or PR−, HER-2 +), and basal-like (ER−, PR−, HER-2−) [2]. Diagnosis of breast cancer through early screening enables complete treatment at an early stage and increases the survival rate [1,3]. Screening methods through liquid biopsy, combined with high-sensitivity molecular detection technology and advanced bioinformatics protocols for metastasis prediction, can greatly improve screening and monitoring at each cancer stage [3,4].

The most actively studied screening and monitoring blood test ctDNA was analyzed based on the fact that solid tumors are characterized by a plethora of genomic abnormalities. Recently, GRAIL has demonstrated a moderate sensitivity of <55% at a specificity >99% with high-precision information on the tissue of origin through targeted methylation assays for more than 50 cancer types. However, the sensitivity for early detection of breast cancer is still as low as 30% [5].

To overcome the low sensitivity of blood tests using ctDNA in breast cancer, we established a strategy for the early detection of breast cancer through blood-cell-based circulating transcriptome blood tests: (I) Cancer-related mRNA can also be expressed in immune cells because immune cells around cancer cells mimic and interact with tumor cells. Therefore, it is possible to increase the sensitivity of early cancer cells through cancer-related genome studies that are affected not only by tumor cells, but also by immune cells [6]. (II) If oncogenes elevated in cancer are detected even in an environment with abundant immune cells in the blood, it can simplify complex analytical procedures such as the isolation of circulating tumor cells (CTCs) [4,5].

**Figure 1:**
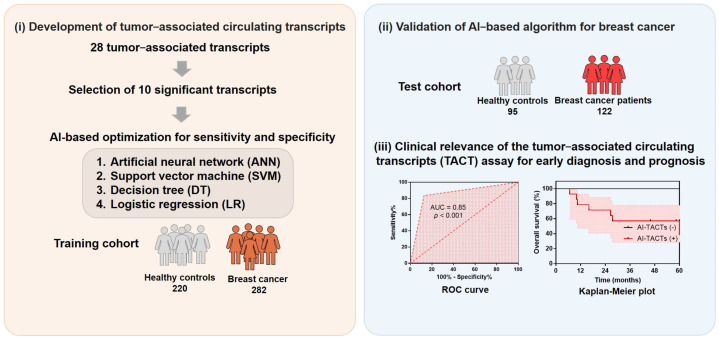
Developmental strategy. Study design for the development and validation of the tumor-associated circulating transcript (TACT) assay.

We previously identified mRNA-based methods involving PCR blood tests for five markers (*EPCAM*, *KRT19*, *ERBB2*, *MKI67*, and *TERT*) as an alternative breast cancer screening tool [7,8,9]. The mRNA genes we selected did not focus on a specific mutation sequence for each individual, but focused on markers with increased genetic expression by limiting to cancer cells and immune cells present in the blood. It is involved in all immune cells and cancer-cell-derived substances that interact in the blood and has useful diagnostic and prognostic information [9]. First, we profiled mRNA in blood samples from breast cancer patients who encompassed heterogeneous cancer subtypes and stages, and then compared their mRNA profiles with those of healthy subjects to identify tumor-associated circulating transcripts (TACTs). Subsequently, we stratified the TACTs using machine learning to develop a highly sensitive predictive model validated with a training set and a subsequent test set, and evaluated the clinical relevance by analyzing the receiver operating characteristic (ROC) curve and the Kaplan–Meier plot for the TACT assay (Figure 1).

## 2. Results

### 2.1. Development of TACTs for Breast Cancer Diagnosis

We profiled the expression of 28 candidate markers using initially designed primers in four breast cancer cell lines representing subtypes: SKBR3 (HER-2 subtype), MDA-MB-231 (basal-like subtype), BT-474 (luminal B subtype), and MCF-7 (luminal A subtype). We then profiled candidate markers in normal and patient populations using small samples as pilot studies. Of the 28 markers, 18 markers that did not show target amplification or did not differ between breast cancer patients and healthy controls and had low expression in breast cancer were excluded (Table S1). We selected ten tumor-associated transcripts (*EPCAM*, *KRT19*, *ERBB2*, *MKI67*, *TERT*, *VIM*, *NPTN*, *MCAM*, *SNAI2*, and *FOXA2*) with high expression that are specifically upregulated in the cellular environment of cancer patients compared with the normal control group. We thought that not only cancer cell-derived mRNA, but also cancer-related mRNA, which is affected by immune cells in the blood that interacts with cancer cells, would be expressed simultaneously. Then, ten tumor-associated transcripts were optimized as highly accurate and sensitive primers of corresponding transcripts and validated with four representative breast cancer cell lines SKBR3, BT-474, MDA-MB-231, and MCF-7 spiked in normal blood. The cancer cells were serially diluted 10-fold from 1 × 10^6^ to 1 × 10^0^ cells/mL, and the last serial linear concentration that produced three positive replicates was identified. The overall detection limits of this RT-qPCR assay for the 10 tumor-associated transcripts developed ranged from 10^1^ to 10^0^ cells/mL, indicating high sensitivity (Figure S1).

### 2.2. Assessment for Performance of TACTs in Patients with Breast Cancer versus Healthy Controls

We prospectively recruited 404 patients with stage I–IV breast cancer and 315 healthy controls (Table 1). Blood samples from patients were organized according to cancer stage and subtype. Samples from healthy controls were organized based on the proportion of ages with breast cancer prevalence. The assay was double-blinded and validated. Details on the study design are described in Figure 1.

### 2.3. Predictive Modeling of TACTs Using the Training Set and Test Set

**Figure 2:**
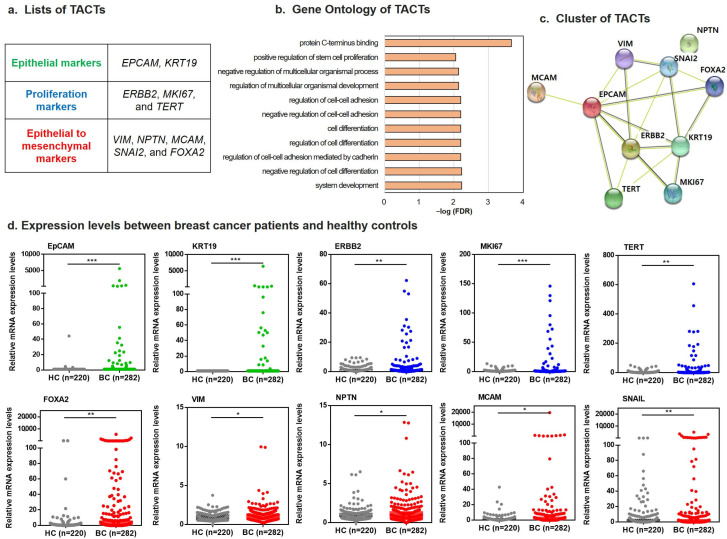
Identification of TACTs. (**a**) Classification of 10 significant transcripts according to epithelial, proliferation, and epithelial-to-mesenchymal features. (**b**) Gene Ontology analysis of TACTs to determine biological and molecular function. (**c**) Interaction network of the 10 TACTs. (**d**) mRNA expression levels of each TACT in healthy controls (HCs) and breast cancer patients (BC). * *p* < 0.05, ** *p* < 0.01, *** *p* < 0.001.

The TACTs represented three biological processes: epithelial origin (*EPCAM* and *KRT19*), proliferation (*ERBB2*, *MKI67*, and *TERT*), and epithelial-to-mesenchymal transition (EMT; *VIM*, *NPTN*, *MCAM*, *SNAI2*, and *FOXA2*) (Figure 2a). Gene Ontology (GO) revealed that all 10 are involved in protein c-terminal binding, positive regulation of stem cell proliferation, cell–cell adhesion regulation, and cell differentiation. These functions imply an association with cancer development (Figure 2b). Additionally, our interaction network analysis indicated that all transcripts except NPTN were connected (Figure 2c).

In vitro diagnostics showed that the individual 10 transcripts were significantly higher in breast cancer patients than in healthy controls (all *p* < 0.01) and showed clinical relevance of TNM stages and subtypes (Figure 2d, Figures S2 and S3), but the sensitivity of each individual marker was too low to distinguish between normal and cancer patients. In addition, the characteristics of individual markers expressed in each patient were different. An integrated understanding of each transcript for an individual patient is required to increase the sensitivity of early diagnosis. Therefore, we combined each marker to investigate the clinical relevance of these TACTs.

To optimize TACTs as early predictors of breast cancer, we applied four different machine-learning models (decision tree (DT), logistic regression (LR), support vector machine (SVM), and artificial neural network (ANN)) to the relative mRNA expression of 10 TACTs. We selected the classifier with the highest accuracy after 1000 iterations. The best model was then chosen after processing with a training set (282 breast cancer patients and 220 healthy controls) and a test set (randomizing control and cancer patient samples).

**Figure 3:**
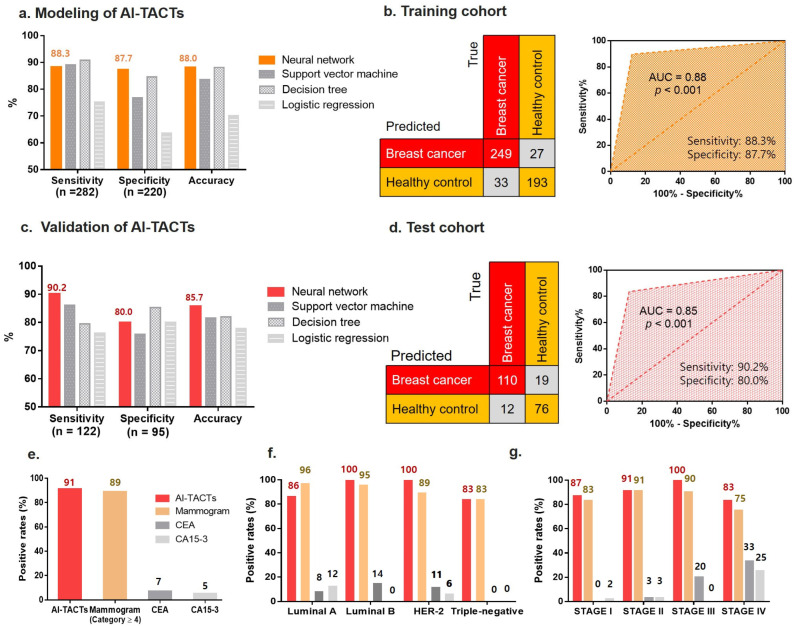
Modeling classifiers and validation of the TACT assay in healthy controls and breast cancer patients. (**a**) Optimization of artificial intelligence analyses (decision tree (DT), logistic regression (LR), artificial neural network (ANN), and support vector machine (SVM)) to model TACTs for distinguishing breast cancer patients from healthy controls. (**b**) Sensitivity and specificity of the ANN model in the training set. (**c**) Validation of TACTs using optimized DT, LR, ANN, and SVM. (**d**) Sensitivity and specificity of the ANN model in the test set. (**e**) Comparison of breast cancer positivity rates between the TACT assay and conventional breast cancer diagnostic methods (i.e., mammograms and CEA/CA15-3 blood markers). (**f**) Sensitivity of the TACT assay and conventional diagnostic methods for differentiating breast cancer subtypes. (**g**) Sensitivity of the TACT assay and conventional diagnostic methods for differentiating breast cancer stages. AUC, area under the receiver operating characteristic (ROC) curve.

To select the best model, we determined their sensitivity, specificity, and accuracy (positive predictive value (PPV) and negative predictive value (NPV)) (Figure 3a). Using the training set, the ANN model had 88.3% sensitivity, 87.7% specificity, and 88.0% accuracy (*p* < 0.001) (Figure 3b and Table S2). Using the test set (122 breast cancer patients and 95 healthy controls), the ANN model had superior sensitivity (90.2%), specificity (80.0%), and accuracy (85.7%) compared with the other three models (Figure 3c,d) (*p* < 0.001). These results indicate that the model is not overfitted and has moderate diagnostic value.

### 2.4. Diagnostic Performance of TACTs According to Breast Cancer Stage

Relative to the value of 90.2% achieved using the TACT assay on our test set, the sensitivity values of other conventional assays (mammogram, CEA, and CA 15-3) were comparable or much lower, at 89%, 7%, and 5%, respectively (Figure 3e). To gain the detailed clinical relevance of subtypes and stages in breast cancer, we analyzed the sensitivity of TACTs in each breast cancer subtype. The sensitivity values of the TACT assay by breast cancer subtype were 86%, 100%, 100%, and 83% for luminal A, luminal B, HER-2, and triple-negative breast cancer, respectively, and the sensitivity of the assay for these subtypes corresponded to that of the mammogram (96% for luminal A, 100% for luminal B, 100% for HER-2, and 83% for triple-negative breast cancer). Moreover, compared with the sensitivity values of conventional assays for the blood markers CEA (8% for luminal A, 14% for luminal B, 11% for HER-2, and 0% for triple-negative breast cancer) and CA 15-3 (12% for luminal A, 0% for luminal B, 11% for HER-2, and 0% for triple-negative breast cancer), this showed that the sensitivity of the TACT assay was remarkably high (Figure 3f). By cancer stage, the sensitivity values of the TACT assay were 87%, 91%, 100%, and 83% for patients with stage I (early-stage), stage II, stage III, and stage IV breast cancer, respectively (Figure 3g). Thus, the assay appears capable of detecting early-stage breast cancer.

### 2.5. Predictive Ability of the TACT Assay for Poor Prognosis in Metastatic Breast Cancers

**Figure 4:**
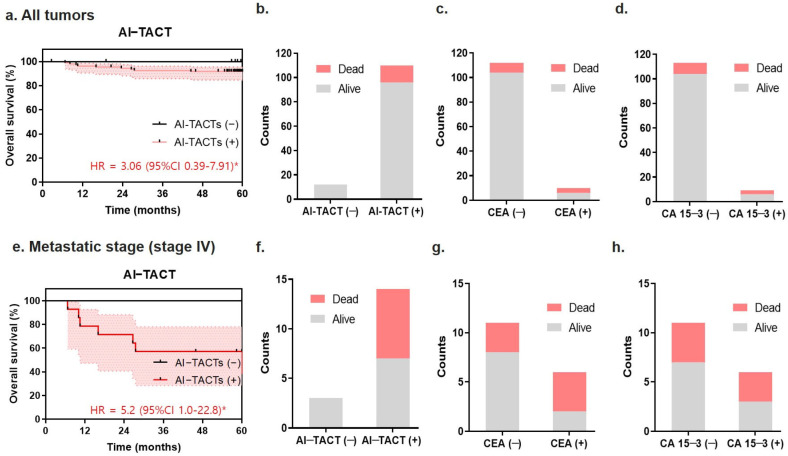
Prognostic value of the TACT assay in breast cancer patients. (**a**) Five-year overall survival of all samples in the test cohort (*n* = 122) according to the TACT assay. (**b**) Survival status of patients in the test cohort according to the AI-TACT assay. AI-TACT (−) included AI-TACT (−) to AI-TACT (−) and TACT (+) to AI-TACT (−) before and after treatment. AI-TACT (+) included AI-TACT (−) to AI-TACT (+) and TACT (+) to AI-TACT (+) before and after treatment. (**c**) Survival status of all samples in the test cohort according to CEA. (**d**) Survival status of all samples in the test cohort according to CA15-3. (**e**) Five-year overall survival of patients with metastatic tumors in the test cohort according to the AI-TACT assay (*n* = 17). (**f**) Survival status of patients with metastatic tumors in the test cohort according to the AI-TACT assay. (**g**) Survival status of patients with metastatic tumors in the test cohort according to CEA. (**h**) Survival status of patients with metastatic tumors in the test cohort according to CA15-3. Log-rank test. * *p* < 0.05.

To analyze the prognostic performance of the TACT assay, the association between TACTs and overall survival (OS) was investigated. Using Kaplan–Meier analysis and log-rank test, the positivity of the TACT assay resulted in poor survival outcomes (HR = 3.06 (0.39–7.91), *p* < 0.05). Breast cancer blood samples with positive TACTs had a threefold higher risk of poor prognosis than samples with negative TACTs (Figure 4a). The TACT assay predicted the outcomes of all 12 patients who died (9.8% out of 122 patients) during the 5 years after breast cancer diagnosis (Figure 4b). Conventional prognostic markers (i.e., CEA or CA15-3) were less accurate in predicting poor prognosis among those 12 patients (Figure 4c,d). When patients were stratified by treatment, positive TACT was related to a higher propensity for distant metastasis and the triple-negative subtype (Figure S4). Seven of the 14 patients (50%) with positive TACT results died during the 5 years after metastatic diagnosis (HR = 5.2, 95% CI 1.0–22.8, *p* < 0.05), whereas patients with negative TACT survived, supporting the association between prognosis and the ability of this indicator to predict metastasis (Figure 4e,f). By contrast, the use of CEA or CA15-3 markers did not yield a significant association between the negative and positive markers in patients with metastatic disease (Figure 4g,h).

## 3. Discussion

Through the early detection of breast cancer, surgical treatment can be used to completely remove malignant tumors and reduce mortality by assigning various treatment options to the patient. Mammography, which is the most commonly used breast cancer screening test, is known to have a sensitivity of 66% and a specificity of 92% [10]. However, recent studies have shown that breast mammography tests do not contribute to mortality reduction and have a false-positive rate of over 30%. Sixty-five percent of false-positive results lead to unnecessary surgical procedures and patient anxiety [11].

The measurement of blood tumor markers is used to detect early signs of cancer through screening or for monitoring a patient’s cancer status during or after chemotherapy. Serum tumor markers such as carcinoembryonic antigen (CEA) and cancer antigen 15-3 (CA 15-3) have been used for cancer screening, but none of the currently tested markers are suitable for screening the entire population because they have a low specificity. Moreover, these markers have been reported to have a low sensitivity for the detection of early breast cancer [12].

Blood-based tests in the circulation can complement existing diagnostic tools to improve the performance of breast cancer screening and detection. Among the blood-based tests, the existing CTC method has the advantage of knowing the origin of cancer because tumor cells are generated from cancer, but there is a limit to improving the test performance because of the small number of CTCs that can be isolated from early-stage cancers. Our assay can help to improve sensitivity for cancer-related mRNA expression in cancer environments by differentiating the cancer cell environment from the normal cellular environment [13]. In several studies, the mRNA markers we profiled were already reported to be highly expressed in tumor tissues [14,15,16,17,18]. We found that these cancer-associated markers were also highly expressed in blood.

We profiled 28 tumor-associated transcript known markers to develop and validate the highly sensitive and specific non-invasive biomarkers of the early detection and monitoring of breast cancer. We excluded markers that did not show amplification of targets, did not differ between breast cancer patients and healthy controls, and had low expression in breast cancer. These criteria yield 10 TACTs. To establish the functional features of the 10 TACTs, GO analysis was performed using the Database for Annotation, Visualization, and Integrated Discovery (DAVID). The TACTs are involved protein C-terminus binding, positive regulation of stem cell proliferation, cell–cell adhesion regulation, and cell differentiation, which are associated with cancer development.

The introduction of TACTs into blood tests is an easy-access diagnosis method that enables early detection of breast cancer in a high-risk population as well as tentative prognosis of the metastatic stage. Supplementary diagnostic tools associated with this assay also help us better understand individual patients based on the molecular features of an aggressive tumor. Early cancer detection can reduce treatment costs by USD 26 billion annually [19,20,21], and the TACT assay is applicable to a wide age range of women.

Our study focused on the expression of cancer-associated transcripts expressed in all cells present in the blood. Among the various immune cells, there are immune cells that increase in close association with tumors. M2-like tumor-associated macrophages are attributed to bone-marrow-derived progenitor cells and Tregs in peripheral blood, and higher numbers of Tregs have been reported in the peripheral blood of breast cancer patients compared with healthy controls. In addition, an increase in dendritic cells was also observed in the peripheral blood of breast cancer patients, with higher levels in HER-2-positive breast cancer patients than in HER-2-negative patients, suggesting a difference between the various breast cancer subtypes. It has been reported that various cancer-related genes (*EpCAM*, *VIM*, and *NPTN*, among others) increase in these immune cells. Therefore, our study, as a baseline study, can be a solution as an assay for early diagnosis by comparing the complex expression of immune cells and cancer cells with markers expressed in normal blood [22,23].

Next-generation sequencing (NGS)-based investigations are drawing considerable attention because they provide massive amounts of information, but their clinical use is limited by cost and reproducibility issues [24,25]. Our assay is less expensive and has higher throughput than NGS-based testing [25,26]. In addition, our mRNA-based breast cancer diagnostic method, which can quantify mRNA relevant to active tumor-driver genes in real time, has several advantages over DNA for gene expression, including the possibility that it lacks any requirement for nuclear transcription or localization. Moreover, the mRNA regions harbor prognostically useful information [7,9,26,27], because mRNA is made from DNA through a transcription process to be translated into protein, and it is the most easily understood source of nucleic acids for the current state of cancer. Our newly developed mRNA assay was designed using the clinically related coding mRNA region, which is highly expressed in breast cancer patients. In order to secure the expression of mRNA, which is fragile compared with the structure of the DNA, the length was designed to be within 100 bp to minimize degradation, and validated the specificity.

As a result of this study, the overall sensitivity and specificity of breast cancer diagnosis using TACT markers were found to be 90.2% and 80.0%, respectively (Figure 3) The sensitivity and specificity of various screening and imaging tests were compared with the TACT assay to confirm the clinical usefulness for breast cancer diagnosis [28,29,30]. Wang et al., demonstrated that the sensitivity and specificity of the CEA and CA15-3 were 22.6 to 51.6% and 94.3 to 97.1%, respectively [31]. Shao et al., reported that the sensitivities of CEA analysis for cancer detection were 4.6%, 11.4%, and 15.4% for breast cancer stages I, II, and III, respectively. Moreover, the sensitivities of CA 15-3 were 5.6%, 16.9%, and 17.1% for breast cancer stages I, II, and III, respectively [32]. Breast cancer patients in the same cohort were analyzed. The sensitivity of CEA was 6.6% and that of CA15-3 was 5.6%. In the case of mammography, Bone et al., reported a sensitivity of 83.9% and a specificity of 63.3% in 81 patients with breast cancer and 30 healthy subjects [33]. Malur et al., reported that 155 of 235 patients were positive (sensitivity of 65.9%) and 144 of 204 healthy controls were negative (specificity of 70.6%) [34]. The sensitivity of the TACT assay was significantly higher than the sensitivity of the standard blood tumor marker assays and mammography.

Despite tremendous advances in the treatment of breast cancer, the morbidity and mortality rates of advanced and metastatic breast cancer remain high at about 50% to date [35,36]. In general, the recurrence and prognosis of breast cancer patients are influenced by various factors such as age, sex, TNM stage, subtype, and treatment modality. Our study investigated whether the prognosis was affected by positive or negative AI-TACT. It showed poor prognosis, especially when subdivided into stage IV. We believe that our study may jointly see the prognostic potential as an adjuvant to the prognostic biomarkers of CEA and CA15-3. The size of the tumor, evaluated as the largest diameter of the tumor, is fundamental for TNM staging, and it is an important determinant for predicting a poor prognosis as the stage of TNM increases, but it is evaluated as a tumor [37,38,39]. Our study has the advantage that these tissue prognostic observations can be measured through blood.

In this study, we also demonstrated that patients in the positive TACTs group had a significantly shorter OS than those in the negative TACTs group (Figure 4). The patients who tested positive for TACT had three- and fivefold higher risk in all stages and the metastatic stage, respectively, compared with the patients who tested negative for TACT.

Taken together, the present study suggests that the detection of TACTs in the blood might provide a better estimate of the risk of relapse and facilitate individualization of treatment.

Despite the validation of rigorously designed breast cancer patients and healthy controls considering the prevalence of ages, we still need to improve assay specificity and extend multi-cross-validation. A further study would be helpful to understand the crosstalk between the tumor microenvironment and circulating cells by analyzing the expression of TACT in pairs between tumor tissues and blood samples using immunofluorescence or immunohistochemistry in future studies. Moreover, long-term randomized trials with follow-up of healthy controls and prognostic values in breast cancer patients will be prospectively analyzed considering age, pregnancies, menopause, and the use of oral contraceptive therapies.

## 4. Materials and Methods

### 4.1. Cell Lines and Cell Culture

Four cell lines representing subtypes of breast cancer (SK-BR-3, MCF-7, BT-474, and MDA-MB-231) were used for initial primer development and gene-specific quantification. The four selected breast cancer cell lines have various protein expression characteristics of breast cancer: SKBR3 (human epidermal growth factor receptor 2 (HER-2) overexpression and weak expression of estrogen receptor (ER) and progesterone receptor (PR)), MCF-7 (overexpression of ER and PR and weak expression of HER-2), MDA-MB-231 (HER, weak expression of ER and PR), and BT-474 (overexpression of HER-2 and ER). SK-BR3 (KCLB No. 30030), MCF-7 (KCLB No. 30022), BT-474 (KCLB No. 60062), and MDA-MB-231 (KCLB No. 30026) were purchased from Korea Cell Line Bank (Seoul, Korea)). SKBR-3, MCF-7, BT-474, and MDA-MB-231 cell lines were grown at 37 °C in a humidified 5% CO incubator. SK-BR-3, MCF-7, BT-474, and MDA-MB-231 cells were cultured in RPMI 1640 medium (Gibco-BRL, Carlsbad, CA, USA), and all media were added with 10% fetal bovine serum (FBS), penicillin at 100 U/mL, and streptomycin at 100 μg/mL (Gib-co-BRL) before use.

### 4.2. Selection and Blood Spiking Test of Tumor-Associated Circulating Transcripts

We selected 10 tumor-associated circulating transcripts representative of the epithelium (*EPCAM* and *KRT19*), proliferation (*ERBB2*, *MKI67*, and *TERT*), and EMT (*VIM*, *NPTN*, *MCAM*, *SNAI2*, and *FOXA2*), and optimized highly accurate and sensitive primers of corresponding transcripts (Figure S1). To find out whether RT-qPCR of ten TACT markers could detect the breast cancer cells in blood, SK-BR-3, MCF-7, MDA-MB-231, and BT-474 cells were spiked in 7.5 mL of blood. The breast cancer cell lines were mixed with blood from a total of 1 × 10^5^ cells to 1 cell, and the cDNA prepared from each sample were subjected to the assay.

### 4.3. Patient Cohorts

A total of 404 types of blood were collected from patients over 20 years who were diagnosed with breast cancer. For healthy controls, 315 blood samples were obtained from women without a breast cancer history. All of the blood was drawn before the treatment and followed sequential screening after the treatment (Figure S5). All of the patients were followed up for at least 5 years. These blood samples were collected after receiving written consent from the Institutional Ethics Committee of Yonsei Severance Hospital. The approval number for breast cancer patients is 1-2010-0018, and the approval number for healthy controls is 4-2011-0011. The healthy control group was additionally evaluated with a sample that received written consent from Yonsei University Wonju University (approval number 1041849-201311-BM-020).

### 4.4. Peripheral Blood from Patients and Healthy Controls

After withdrawing the first 5 mL of blood from each participant, it was discarded for reducing epithelial contamination, and then 7.5 mL of blood was withdrawn for the experiments. The blood sample was prepared as discussed in our previous reports [7,8]. Briefly, ACK solution (0.15 M NH4Cl, 1 mM KHCO3, and 0.1 mM Na2EDTA) was added to lyse the red blood cells in blood, and RNA was isolated using Isol-RNA Lysis Reagent (5 Prime, Austin, TX, USA), according to the manufacturers’ instructions. All of the processes were performed within 4 h. The purity and concentration of total RNA were confirmed using an Infinite 200 ^®^ (Tecan, Salzburg, Austria). All steps were performed under RNase-free conditions. The isolated total RNA was stored at −70 °C.

### 4.5. Reverse Transcription-Quantitative PCR Assay

Complementary DNA (cDNA) was synthesized using the M-MLV Reverse Transcriptase enzyme (Invitrogen, Carlsbad, CA, USA) and a random hexamer (Invitrogen). The CFX-96 PCR system (Bio-Rad, Hercules, CA, USA), which performs real-time quantification, detected fluorescence using TaqMan probes designed in house. The assay includes 10 µL of 2 × Thunderbird probe qPCR mixture (Toyobo, Osaka, Japan), 5 µL of primer and TaqMan probe mixture, and 2 µL of template cDNA and distilled water (D.W.). Positive and negative controls were used throughout all experimental procedures. Each sample was tested in duplicate. The reaction process was repeated 40 times, after 3 min at 95 °C, 15 s at 95 °C, and 30 s at 55 °C.

Gene expression used the comparative Ct method (ΔΔCt method) and normalized to the internal housekeeping gene GAPDH. The amount of target relative to the calibrator was given as 2 − ΔΔCt, and the analysis used the following equation: ΔΔCt = [ΔCt(test) = Ct) − Ct(reference test)] − [ΔCt(calibrator) = Ct(target calibrator) − Ct(reference calibrator)].

### 4.6. Sensitivity and Specificity by Deep Learning Analysis

The TACTs were clinically evaluated using four types of machine learning classifiers, namely decision tree (DT), logistic regression (LR), artificial neural network (ANN), and support vector machine (SVM). All classifiers were implemented using R package caret (method “rpart” for DT, “glm” for LR, “nnet” for ANN, and “e1071” for SVM). The classifier with the best accuracy over 1000 iterations was selected. The best model of the algorithm was considered based on acceptable sensitivity, specificity, and accuracy.

Each deep learning model was processed using two sets: the training set and test set. For the training and validation of the model using artificial intelligence analysis, 70% of the total subjects, including 282 patients with breast cancer and 220 healthy volunteers, were used (the training set), and the model was created. The remaining 30% of the total population, including 122 patients with breast cancer and 95 healthy volunteers, was used to test the model.

The selected artificial neural network (ANN) is a computer model made up of highly interconnected nodes, an adaptive algorithm that can learn from pattern to pattern, and is a proper method for medical use [40]. An ANN estimates the impact on input variables and outcomes by increasing or decreasing the value of the connection weights between nodes through “learning”. Specifically, its ability to help predict outcomes is determined by the connections between neurons in the ANN [41]. Numerous studies have verified that the ANN model could categorize breast cancer patients using medical images [42,43]. Moreover, the ANN model has been used for the analyses of genomic data [44]. The ANN model used in this study was developed as a three-layer, feed-forward method. The layers of the ANN model consist of an input layer of 10 TACT expression levels, a hidden layer with 1000 hidden nodes, and an output layer with a single node generating breast cancer positivity.

### 4.7. Bioinformatic Analysis of TACTs

GO enrichment analysis of TACTs genes was implemented using the Database for Annotation, Visualization, and Integrated Discovery (DAVID) tool. The GO term was displayed with *p* < 0.05. Then, we used the STRING database (https://string-db.org/ accessed on 20 June 2022) to analyze and visualize the protein–protein interaction (PPI) of the TACTs.

### 4.8. Statistical Analysis

Graphs were presented using GraphPad Prism software version 7.00 (GraphPad, La Jolla, CA, USA), and statistical analysis was performed using Statistical Package for the Social Sciences (SPSS) software version 18.0 (SPSS Inc., Chicago, IL, USA). A cutoff analysis of each marker to differentiate between normal and cancer patients performed ROC curve analysis. Two-group comparisons and multiple-group comparisons were performed by Student’s *t*-test, and correlation analysis was performed by Pearson correlation and one-way ANOVA. The sensitivity, specificity, and accuracy determined the optimal diagnostic assay. Overall survival (OS) was analyzed using the Kaplan–Meier method, and statistical significance was compared between groups using the log-rank test. Finally, a Cox regression model was used to evaluate the association between OS for positive and negative TACT. A *p*-value < 0.05 was considered statistically significant.

## 5. Conclusions

In conclusion, this study is the first to demonstrate that the TACT assay is a cost-effective method for making risk-stratified predictions using mRNA-based blood tests combined with neural networks. This novel assay can provide essential data that facilitate early and appropriate therapeutic decisions.

## Figures and Tables

**Table 1:** Clinicopathologic characteristics of breast cancer patients.

Cohorts	Training Cohort, *n* (%)		Test Cohort, *n* (%)	
Variable	Healthy Control (*n* = 220)	Breast Cancer (*n* = 282)	Healthy Control (*n* = 95)	Breast Cancer (*n* = 122)
Age at diagnosis				
<50 years	174 (79.1)	157 (55.7)	66 (69.5)	61 (50)
≥50 years	46 (20.9)	125 (44.3)	29 (30.5)	61 (50)
TNM stage				
I		126 (44.7)		47 (38.6)
II		66 (23.4)		32 (26.2)
III		14 (5.0)		10 (8.2)
IV		24 (8.5)		17 (13.9)
Unknown		52 (18.4)		16 (13.1)
Therapy				
Adjuvant		210 (74.5)		92 (75.4)
Neoadjuvant		48 (17.0)		13 (10.7)
Metastasis		24 (8.5)		17 (13.9)
Subtypes				
Luminal A		165 (58.5)		65 (53.3)
Luminal B		30 (10.6)		21 (17.2)
HER-2		38 (13.5)		18 (14.8)
Triple-negative		45 (16.0)		18 (14.8)
Unknown		4 (1.4)		0 (0)
CEA				
Positive		26 (9.2)		10 (8.2)
Negative		256 (90.8)		112 (91.8)
CA15-3				
Positive		19 (6.7)		9 (7.4)
Negative		263 (93.3)		113 (92.6)
Survival status				
Alive		242 (85.8)		110 (90.2)
Dead		40 (14.2)		12 (9.8)
Mammography (category)				
<4		19 (6.7)		8 (6.6)
≥4		263 (93.3)		104 (85.2)

## Data Availability

Not applicable.

## Supplementary Materials

The following supporting information can be downloaded at https://www.mdpi.com/article/10.3390/ijms23169140/s1.
