# Supplementary material for: Blood Test for Breast Cancer Screening through the Detection of Tumor-Associated Circulating Transcripts
Source: Int J Mol Sci. 2022 Aug 15;23(16):9140. doi: 10.3390/ijms23169140 (PMC9409068; doi:10.3390/ijms23169140)
Supplement: Supplementary file 1 [file ijms-23-09140-s001.zip › ijms-1830277-supplementary.pdf]

Article

Blood Test for Breast Cancer Screening through the Detection of Tumor-Associated Circulating Transcripts

Sunyoung Park[1,†], Sungwoo Ahn[1,†], Jee Ye Kim[2], Jungho Kim[3], Hyun Ju Han[4], Dasom Hwang[1], Jungmin Park[2], Hyung Seok Park[2], Seho Park[2], Gun Min Kim[5], Joohyuk Sohn[5], Joon Jeong[6], Yong Uk Song[7], Hyeyoung Lee[1,*], Seung Il Kim[2,*]

Table S1. The lists of profiled 28 genes for selection of TACTs

GENE ID	No of Pilot study (Clinical validation)	Characteristics of expression levels	p value
EPCAM	Breast cancer (282), healthy control (220)	Increase in BC	< 0.05
KRT19	Breast cancer (282), healthy control (220)	Increase in BC	< 0.05
ERBB2	Breast cancer (282), healthy control (220)	Increase in BC	< 0.05
MKI67	Breast cancer (282), healthy control (220)	Increase in BC	< 0.05
TERT	Breast cancer (282), healthy control (220)	Increase in BC	< 0.05
VIM	Breast cancer (282), healthy control (220)	Increase in BC	< 0.05
SNAI2	Breast cancer (282), healthy control (220)	Increase in BC	< 0.05
FOXA2	Breast cancer (282), healthy control (220)	Increase in BC	< 0.05
MCAM	Breast cancer (282), healthy control (220)	Increase in BC	< 0.05
NPTN	Breast cancer (282), healthy control (220)	Increase in BC	< 0.05
TWIST1	Breast cancer (43), healthy control (4)	Decrease in BCs	<0.05
ZEB2	Breast cancer (30), healthy control (40)	Decrease in BCs	0.001
CXCR4	Breast cancer (12), healthy control (4)	No significant difference	0.21
SNAI1	Breast cancer (43), healthy control (4)	Decrease in BCs	< 0.05
CCND1	Breast cancer (43), healthy control (4)	Decrease in BCs	< 0.05
BMI1	Breast cancer (15), healthy control (20)	No significant difference	0.14
MAGEA3	Breast cancer (4), healthy control (30)	Decrease in BCs	< 0.05
PTEN	Breast cancer (43), healthy control (4)	No significant difference	0.86
PROMI	Breast cancer (12), healthy control (4)	No significant difference	0.2
FOXA1	Breast cancer (12), healthy control (4)	No amplification of targets	-
CDH1	Breast cancer (12), healthy control (4)	No amplification of targets	-
CDH2	Breast cancer (43), healthy control (4)	No significant difference	0.71
RRMI	Breast cancer (43), healthy control (4)	Decrease in BCs	< 0.05
CRYAB	Breast cancer (12), healthy control (4)	No amplification of targets	-
KL	Breast cancer (12), healthy control (4)	No amplification of targets	-
SCGB2A2	Breast cancer (72), healthy control (19)	No significant difference	0.4
TFF1	Breast cancer (12), healthy control (4)	No amplification of targets	-
MUC1	Breast cancer (72), healthy control (19)	No significant difference	0.4

Table S2. Sensitivity and specificity of artificial neural network analysis in training and validation sets

Model	ANN		DT		LR		SVM	
	Training Set	Validation Set	Training Set	Validation Set	Training Set	Validation Set	Training Set	Validation Set
Sensitivity	88.3% (95% CI 0.840–0.918)	90.2% (95% CI 0.835–0.948)	90.8% (95% CI 0.868–0.939)	79.5% (95% CI 0.713–0.863)	75.2% (95% CI 0.697–0.801)	76.2% (95% CI 0.677–0.835)	89.0% (95% CI 0.848–0.924)	86.1% (95% CI 0.786–0.917)
Specificity	87.7% (95% CI 0.827–0.918)	80.0% (95% CI 0.705–0.875)	84.6% (95% CI 0.791–0.891)	85.3% (95% CI 0.765–0.917)	63.6% (95% CI 0.569–0.700)	80.0% (95% CI 0.705–0.875)	76.8% (95% CI 0.707–0.822)	75.8% (95% CI 0.659–0.840)
Positive Predictive Value	90.2% (95% CI 0.861–0.935)	85.3% (95% CI 0.780–0.909)	88.3% (95% CI 0.840–0.917)	87.4% (95% CI 0.797–0.929)	72.6% (95% CI 0.671–0.776)	83.0% (95% CI 0.748–0.895)	83.1% (95% CI 0.784–0.872)	82.0% (95% CI 0.743–0.883)
Negative Predictive Value	85.4% (95% CI 0.801–0.897)	86.4% (95% CI 0.774–0.928)	87.7% (95% CI 0.826–0.918)	76.4% (95% CI 0.672–0.841)	66.7% (95% CI 0.599–0.730)	72.4% (95% CI 0.628–0.807)	84.5% (95% CI 0.787–0.892)	80.9% (95% CI 0.712–0.885)

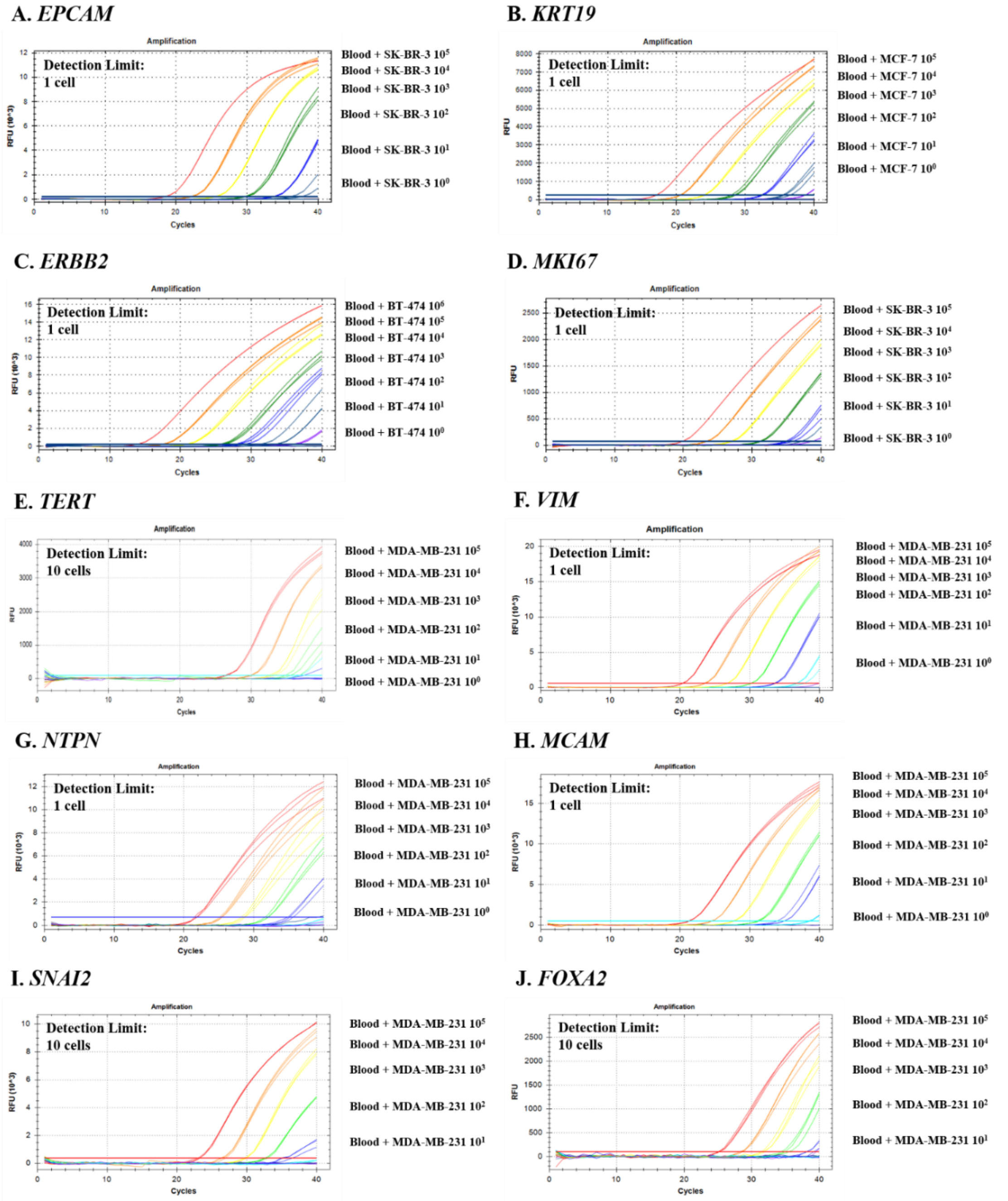

Figure S1. Analytical sensitivity of ten tumor-related transcripts in breast cancer cells. Ten tumor-related transcripts (*EPCAM, KRT19, ERBB2, MKI67, TERT, VIM, NPTN, MCAM, SNAI2*, and *FOXA2*) were designed for highly sensitive detection, and evaluated in four representative breast cancer cell lines SKBR-3, MDA-MB-231, MCF-7, and BT-474. For the analytical sensitivity of RT-qPCR assay, breast cancer cell lines were serially diluted 10-fold from 1 × 10^6 to 1 × 10^0 cells/mL, and the last serial linear concentration that yielded 3 positive replicates was identified. The overall detection limit of this RT-qPCR assay for the ten tumor-related transcripts ranged from 10^1 to 10^0 cells/mL, which indicated high sensitivity. The 10 tumor-related transcripts could be detected in all four representative breast subtypes (luminal A, luminal B, HER2, and triple-negative breast cancer).

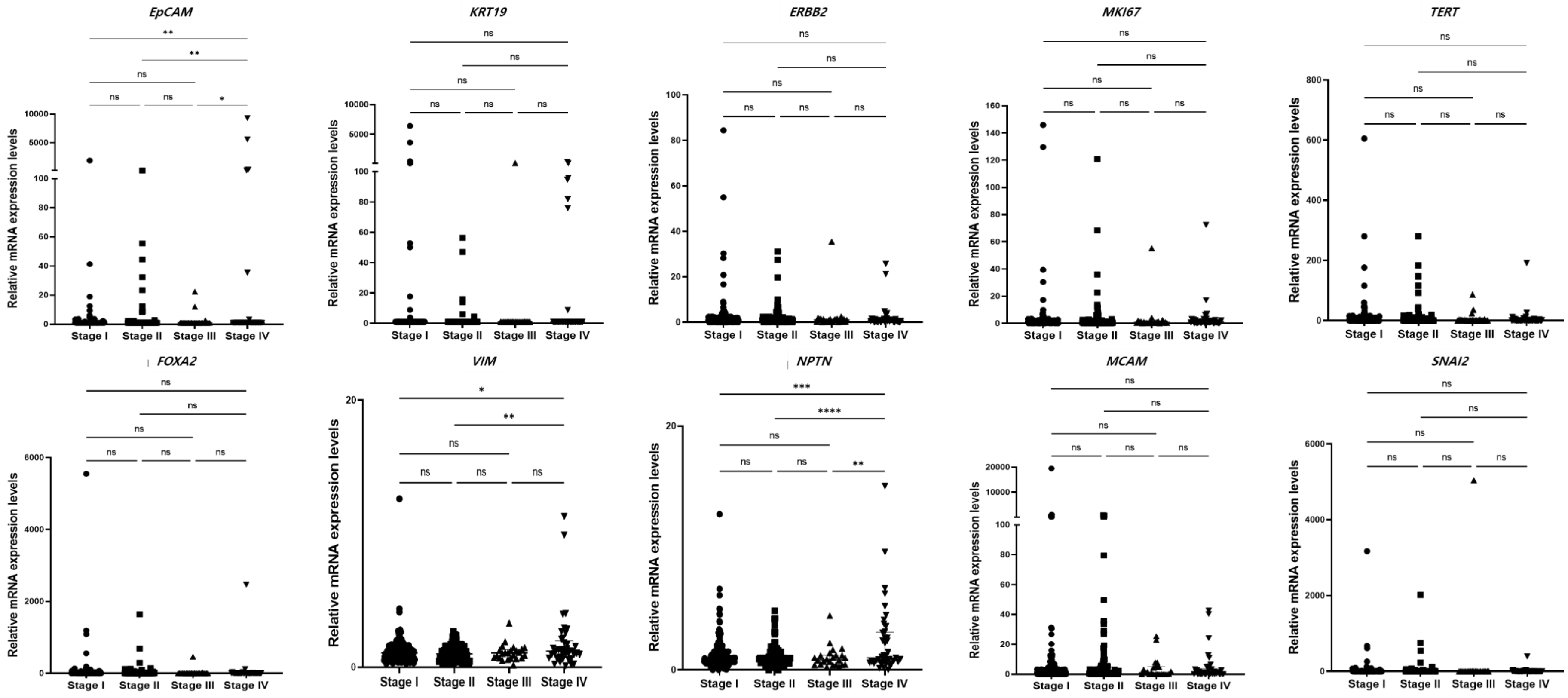

Figure S2. Clinical relevance between each TACT marker and TNM stage. There was statistical difference between stage IV and stage I, stage II in *EpCAM, VIM,* and *NPTN*. In addition, *EpCAM* showed significant difference between stage III compared with stage IV.). *$p < 0.05$, **$p < 0.01$, ***$p < 0.001$. ****$p <$ 0.0001.

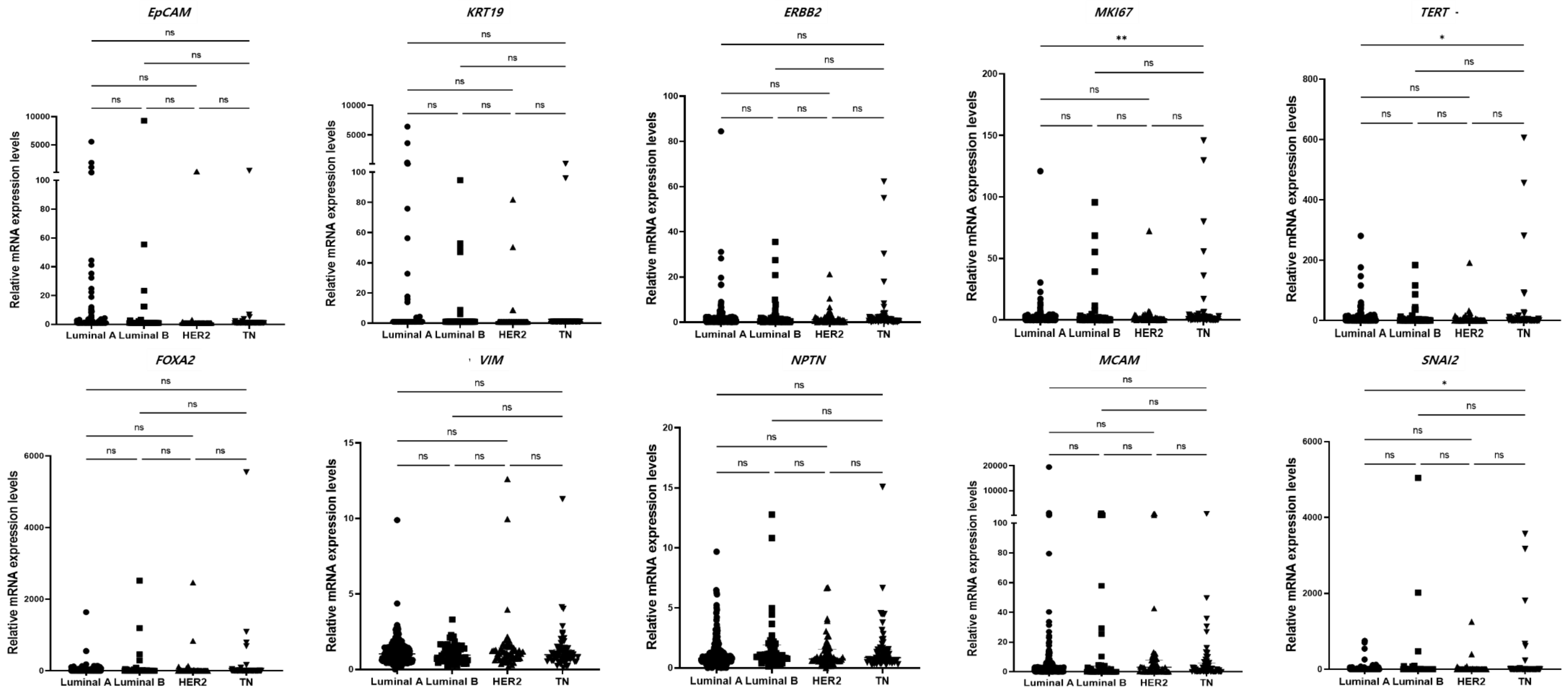

Figure S3. Clinical relevance between each TACT marker and subtypes. *MKI67*, *TERT* and *SNAI2* had significant difference between luminal A and basal-like subtype. *$p < 0.05$, **$p < 0.01$.

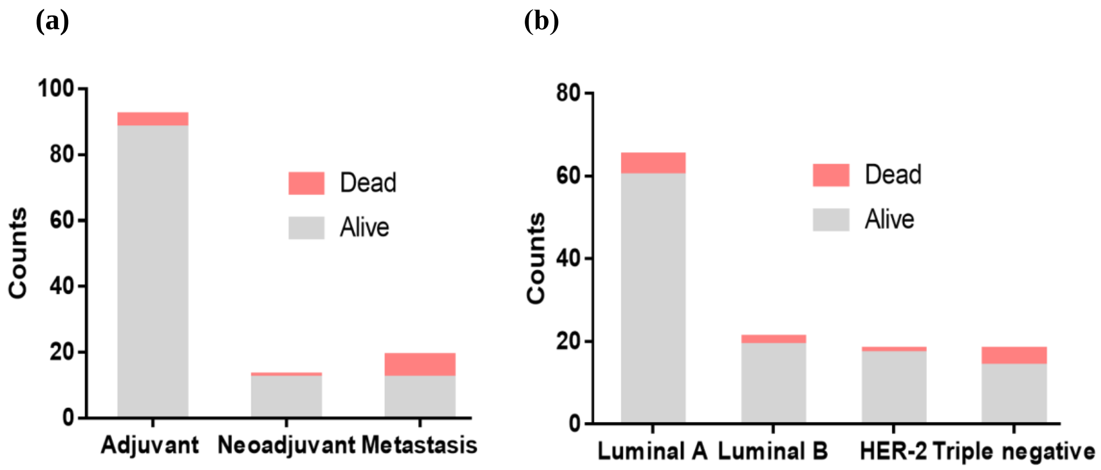

Figure S4. Vital status of stages and subtypes. a. Vital status of adjuvant, neoadjuvant, and metastasis chemotherapy types. b. Vital status of breast cancer subtypes.

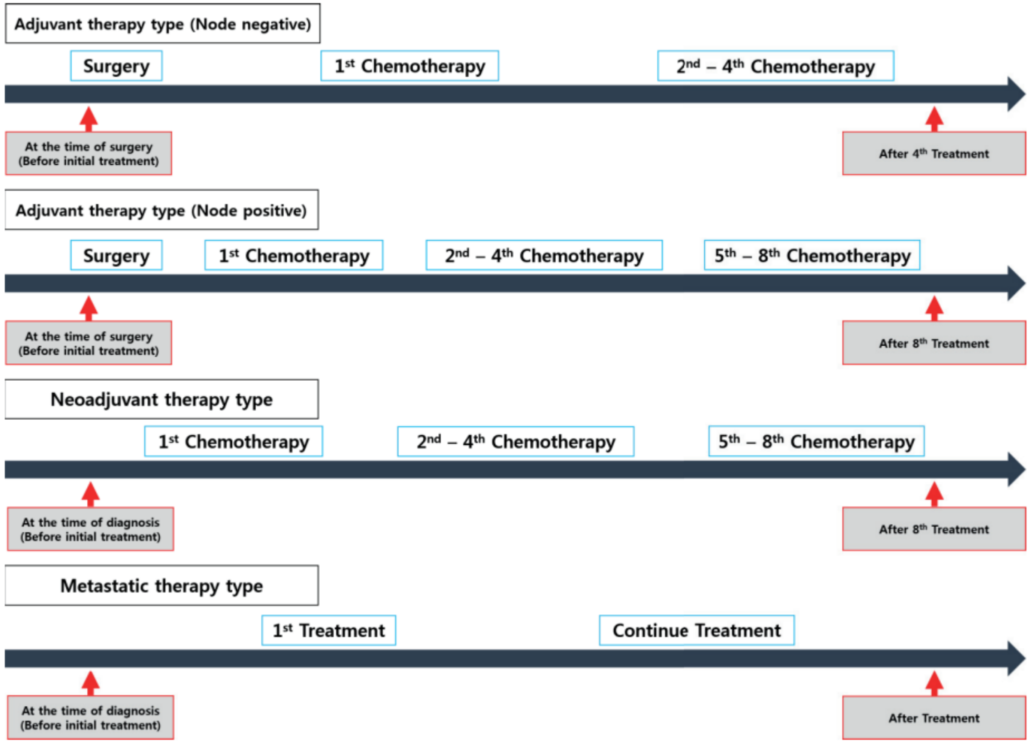

Figure S5. Scheme of adjuvant, neoadjuvant, and metastasis chemotherapy types and blood collection time from the breast cancer patients. Breast cancer patients with the adjuvant setting were treated with up to 4 to 8 chemotherapy, and blood collection was performed after the completion of last chemotherapy. Breast cancer belongs to the neoadjuvant therapy type were treated with 8 chemotherapy, and first blood collection was performed at the time of diagnosis of breast cancer. Breast cancer belongs to metastatic therapy type, the first blood collection was performed at the time of diagnosis, and the last blood collection was performed at the completion of treatment.
